# Neuroexistential psychotherapy: bridging the gap

**DOI:** 10.3389/fpsyg.2026.1805267

**Published:** 2026-06-29

**Authors:** Aldrich Chan, Justin Underwood, Erik Craig, Kirk Schneider, Adam Hauptfeld, Cypress Ledesma, Jason Ouyang, Afrooz Seyedi, Sophia Basso

**Affiliations:** 1 Center for Neuropsychology and Consciousness, Miami, FL, United States; 2 Pepperdine University, Los Angeles, CA, United States; 3 West Los Angeles Veterans Administration, Los Angeles, CA, United States; 4 Center for Existential Studies, Santa Fe, NM, United States; 5 Saybrook University, Pasadena, CA, United States; 6 The Existential-Humanistic Institute, San Francisco, CA, United States; 7 Miami Dade College, Miami, FL, United States; 8 Georgia Southern University, Statesboro, GA, United States

**Keywords:** existential, neuroexistential, neuropsychology, neuroscience and psychotherapy, psychotherapy

## Abstract

As science continues to advance, many psychotherapeutic approaches have updated their theories and practices accordingly. However, existentialism has remained ambivalent to these developments, leading it to appear less effective, rigorous, or valid, despite ample evidence to the contrary. The purpose of this article is twofold. First, it is an invitation for natural-scientifically trained psychologists and psychotherapists to recognize the substantive benefits of grounding their thinking and practice in human-scientific philosophical reflection. Second, to encourage existentially oriented psychologists and psychotherapists to appreciate the added value of natural-scientific perspectives, particularly those that focus on the body and the brain. In short, the overriding aim is to contribute to a more unified epistemological discourse across the disciplines devoted to studying and caring for human beings.

## Introduction

With a recent landmark publication (Hoffman and Lac, 2025), demonstrating robust support for existential therapies as thoroughly consistent with evidence-based practices in psychology, in addition to advances in the neuropsychological sciences, the time is ripe for expanding on their possibilities for a more collaborative approach^1^ to understanding human beings. More specifically, neuropsychology and existential philosophy/psychotherapy, to date, remain relative strangers. At worst, they regard each other with suspicion and criticism; at best, they remain largely inattentive to the fact that both address the same living, breathing, thinking creature: the human being.

The analysis of human existence has been a central concern of Western philosophy since the early 20th century, particularly with the publication of Heidegger's (1962) groundbreaking publication, *Being and Time* (1962/1927), where he first laid out his fundamental ontological analysis of Dasein (*Daseinsanalytik*), his term for the kind of beings we call *human* beings. It was left to two Swiss psychiatrists who knew Heidegger personally and were also deeply immersed in psychoanalytic thought and practice to develop daseinsanalytic approaches to psychiatry, psychopathology, and psychotherapy. Whereas the first, Ludwig Binswanger (1881–1966), was especially influential in articulating daseinsanalytic perspectives in psychiatry and psychopathology, Medard Boss (1903–1990), collaborating with Heidegger himself, developed his own daseinsanalytic approach to psychotherapy (cf. Boss, 1963, 1979; Craig, 1988). While their ideas form the base of modern day Daseinanalysis, the essential principles therein can be found embedded in the foundations of other core existential approaches today, such as existential-phenomenological, Existential Analysis/Logotherapy and Existential-Integrative/Existential-Humanistic (Van Deurzen et al., 2019). Of note, the Existential-Integrative approach, pioneered by Schneider (2008), and endorsed as scientific by Wampold (2007, 2008), offers a holistic approach that is inclusive of the physiological domain, lending itself to be a prime candidate for integration. The major “existentials” according to Boss (1979) include

“the spatio-temporal character of Da-sein, its attunement, its bodyhood, its coexistence…with other people in a shared world, its openness and, finally, the unfolding of inherent potentialities into existential freedom” (p. 199).

Given the intended audience and purposes of this paper, it is important to first provide a brief history of existential philosophy to ensure the reader understands what existential psychology truly encompasses. This will be followed by delineating the relationship between science and existentialism, commonalities in existential narratives, and its overall therapeutic goals. These sections set the foundations for the quest to begin examining how the science of neuropsychology aligns with existential ideas on the human being in relation to space, time, attunement, body, relationship, and freedom. In addition, major topics relevant to psychotherapy such as anxiety, authenticity and courage will be incorporated.

## Brief overview of the history of existential^2^ therap

The word existential comes from the Latin verb *existere*, meaning “to exist,” literally to stand out (ex-ist), appear, or come into being. The adjective, existential, refers to the nature and meaning of *human* existence as such, to what it is to *be* a human being, to live and die, to suffer and rejoice, to love and be loved. Such qualities cannot be grasped by objective, rational, or purely intellectual analysis but only by lived experience.

Some of the earliest renderings of the everyday promises and perplexities of such lived experience appeared in the romantic poetry and literature of writers like Johanne Wolfgang von Goethe (1749–1832), Friedrich Hölderlin (1770–1843), Fyodor Dostoevsky (1821–1881), Henrik Ibsen (1828–1906), and Leo Tolstoy (1828–1910), among others. However, existential philosophy itself began with the works of such proto-existential thinkers as Arthur Schopenhauer (1788–1860), Soren Kierkegaard (1813–1855), and Friedrich Nietzsche (1844–1900). While the above names are relatively familiar in contemporary intellectual culture, two lesser-known 19th-century German thinkers were indispensable for understanding the foundational epistemological issues explored here: Franz Brentano (1838–1917) and Wilhelm Dilthey (1833–1911).

Franz Brentano was a psychologist and pre-phenomenological philosopher of psychology who introduced the idea of intentionality^3^ to 19th and 20th century philosophy. Though hardly a “household name” for existential therapists, his contributions to existential research, thought, and practice were as fascinating as they were significant. Of particular interest for existential thinkers and therapists are his early interest on the meanings of being which awakened Heidegger's life-long fascination with the question of being, his (Brentano's) later publication of *Psychology from an Empirical Standpoint* (1874), and, especially, the fact that both Sigmund Freud (1856–1939) and Edmund Husserl (1859–1938) studied with him at the University of Vienna. Bretano not only introduced the idea of intentionality, which was central to Husserl's development of phenomenology, but also the distinction between genetic psychology and descriptive psychology, with the former being appropriate for the so-called empirical sciences and the latter referring to the study of human consciousness or experience through first person descriptions.

Brentano's contemporary, Wilhelm Dilthey, was a historian, social theorist, psychologist, and hermeneutic philosopher. Like Brentano, who distinguished between genetic and descriptive psychology, Dilthey, a polymath himself, made a decisive contribution by differentiating between natural sciences (*Naturwissenschaften*) and human sciences (*Geisteswissenschaften*; literally, the “sciences of the spirit”). This distinction provides the philosophical and epistemological foundation for our argument here in favor of a more unified science of human existence. For Dilthey the natural sciences, aspiring to establish causal laws, seek to explain (*eklären*) nature, through objective observation, replication, and measurement. In contrast, the human sciences aim to understand (*verstehen*) historically situated, irreplicable, expressions of lived-experience (*Erlebnis*) striving to grasp their meaning. Whereas the natural sciences emphasize explanation and causality, the human sciences emphasize understanding and the interpretive disclosure of meaning. Dilthey summed it up succinctly in his 1883 *Introduction to the Human Sciences*: We explain nature, we understand “the life of the soul (*Seelenleben*).” It was this same distinction that Heidegger conveyed in contrasting calculative thought (*rechnendes Denken*) with contemplative thought (*besinnliches Denken*).^4^

In addition to introducing the distinction between the human and natural sciences, Dilthey was a major figure in the history of hermeneutics, the science and art of human understanding (*verstehen*), an empirical practice one can trace back to the ancient Greeks. Dilthey considered hermeneutics the methodological foundation for the *Geisteswissenschaften* and thought the human being must be considered as both an object, a physical organ^5^ for example, from medical, biological, and neurological perspectives, and as a subject, that is, from psychological, social, mental, moral, and spiritual perspectives. Although the *human* being is an embodied being, it is also a distinctively thinking, feeling, and *choosing* kind of being who is not only conscious of himself or herself but also of *what it means* to be, to suffer and rejoice, to wonder and fear. Animated by the works of Brentano and Dilthey, Heidegger's phenomenological hermeneutic *Daseinsanalytik* considered the human being's capacity for understanding being (*Seinsverständnis*), including its own being, to be the “sole concern of *Being and Time*” (Heidegger, 2001). Thus, for Heidegger, meaning and understanding were not only fundamental ontological characteristics (*existentiale*) of *Dasein*, but the very ones that most fundamentally distinguish the human being's essential character (cf. Craig, 2019, p. 48).

As Craig (2019) pp. 53–54 has also noted, the twentieth century opened with extraordinary ferment for existential philosophy and psychotherapy. Nietzsche died in August of 1900, Freud published the *Interpretation of Dreams* (1900), Husserl introduced phenomenology with his *Logical Investigations* (1900–1901), and Dilthey wrote his influential *The Rise of Hermeneutics* (1900). These and other related events could be said to represent a kind of sea change in the European studies of the mind. However, it was the psychiatrist turned philosopher, Karl Jaspers who was the first to be impressed by Husserl's *Logical Investigations* and apply it to the understanding of psychopathology, a task explicated in his 1913 *General Psychopathology*. Also influenced by Kierkegaard and Nietzsche, he was the first to imagine and articulate a coherent Philosophy of human existence (*Existenzphilosophie*). Eventually, the 20th century saw a number of influential thinkers and writers embraced phenomenology in their thinking and practice, including the philosopher Merleau-Ponty (1908–1961), the philosophers and novelists Jean Paul Sartre (1905–1980), De Beauvoir (1962), Gabriel Marcel (1889–1973), Albert Camus (1913–1960), and Michel Henry (1922–2002), and the psychiatrists Victor von Gebsattel (1883–1976), Eugène Minkowski (1885–1972), Ludwig Binswanger (1881–1966), Medard Boss (1903–1990), Viktor Frankl (1905–1997), R.D. Laing (1927–1989), and Franz Fanon (1925–1961).^6^

### Are science and existential psychology compatible?

Many existential psychotherapists differentiate between the natural sciences and its methods and the human sciences. As May et al. (1958) put it

“it is the endeavor to understand man by cutting below the cleavage between subject and object which has bedeviled western thought and science since shortly after the renaissance.” (p. 11).

Lodged against natural science were arguments against reductionism, objectification, misleading categorizations, and the overall loss of humanity in the process. Paradoxically, it is quite often the case that people forget that science itself is based on a set of philosophical assumptions, for which it would lack purpose and orientation without (e.g., the reality humans perceive can reliably inform us about the nature of reality, reality can be captured through observation and measurement, objective reality is governed by natural laws, etc.). These difficulties are further extended by three core limitations to science:

1) Natural Science does not answer why, only how and what. It can provide no meaning to one's life.
2) Natural Science studies things, not being or the human process of becoming.
3) Through the emphasis of natural science, the treatment of others becomes mechanized and technique focused, which makes it easier to study, but vastly reduces the richness of human experience as individual differences become overlooked.

In contrast, scientifically minded existential psychology is often demeaned for its terminology and ideas that are beyond validation and measurement, making it unreliable and speculative at best. While this may be true for certain concepts inspired by philosophy, the devout existentialist would actually

“aspire to greater empiricism… then that which the natural sciences can achieve [alone]. His prime aim is to adhere to the immediately given objects and phenomena of man's world, to remain with man's undistorted perceptions, and to let the phenomenon speak for themselves and show us their essence and meanings” (Boss, 1963, p. 59).

Thus, any comprehensive system of existence must integrate the ontological with the ontic^7^, the immeasurable with the measurable. Boss further notes

“the very word ‘phenomena,”' is derived from phainesthai, i.e., to shine forth, to appear, unveil itself, come out of concealment or darkness” (p. 28)

Phenomena have multiple layers, with one mode of exploration being the scientific method and another being phenomenology, an approach that cuts through interpretive frameworks and engages with experiences as they present themselves in their raw form. In this context, the phenomenological technique of “bracketing” would only be helpful to scientists; as the importance of evidence often relies on assumptions of the investigator and the audience, including the cultural assumptions about what is meaningful (Wampold, 2007).

May himself was concerned about antiscientific tendencies he saw within some circles of existential psychology (Grogan, 2013). Another champion of the human sciences, Carl Rogers, was the first person to quantitatively study psychotherapy and was presented with the Award for Distinguished Scientific Contributions by the APA (Elkins, 2015). Positivistic methods have complemented existential frameworks. The human sciences should not aim to contradict the natural sciences, yet it has historically existed independently from the natural sciences to better achieve its aim of studying the human psyche (Giorgi, 1970). Science should be a useful tool, not another gateway to yet another true world theory that Nietzsche warned about. Our group believes that a neuroexistential view may bridge this gap.^8^

### Existential narrative

In the traditional existentialist view, human beings are understood as interconnected potentials unfolding over time and space (Boss, 1979/1994). May (1963) described “being” as a “pattern of potentialities” (p. 7). In a way that resonates with basic principles of physics, humans can be seen as coordinated patterns of potential (comprising energy and information) continually actualized through the dimensions of time and space.

One of our main driving forces is to live out our potential. Many conflicts that humans encounter can be conceptualized through this lens. The natural unfolding of potential can stagnate, become restricted, or become chaotically expressed.

“If any organism fails to fulfill its potentialities, it becomes sick, just as your legs would wither if you never walked. But the power of your legs is not all you would lose. The flowing of your blood, your heart action, your whole organism would be weaker.” (May, 1953, p. 66)

Boss (1979/1994) introduces three important questions to ask when understanding the challenges an individual faces:

“(1) How is a person's freedom to carry out his potentialities impaired at any given time? (2) What are these potentialities; and (3) With respect to which entities of the person's world does this impairment occur?” (p. 200)

In addressing these questions contextually, existentialists are interested in Mitwelt (i.e., with world or relationships), Umwelt (i.e., around-world, or body and environment), and Eigenwelt (i.e., world within oneself) (May, 1983, p. 12)—more recently added Uberwelt (spiritual dimension) — (Van Deurzen, 2014).

As examples, humans may experience existential guilt when they do not feel like they are fulfilling their potential; and when severe conditions impose limits on what potentials can be expressed, their personhood narrows to protect themselves. May (1963) elaborates:

“If I opened up, if I communicated, I would lose what little space in life I have” (p. 18).

The individual accepts nonbeing, to preserve what being is left. In contrast, anxiety may be viewed from multiple perspectives, as a

“basic reaction to a danger to his existence, or to some value he identified with his existence” (May, 1953, p. 23)

Or a

“fear of one's own powers, and conflicts that arise from that fear” (May, 1963).

### Therapeutic goal

Boss's (1979) conception of wellbeing is captured by the following descriptions:

“by nature, the human being is a clear, open realm constituted of the ability to perceive and respond, and its task is to allow the presences it perceives to enter into this clearness, and to unfold their meanings and context of reference.” (1979, p. 195)

Thus, the emphasis is

“being-there as an open realm of time in space, my existential potentialities are realized in the way most precisely suited to me, and my existence as an open perceptive responsiveness reaches its greatest range and freedom. As a consequence, my Da-sein is at this moment attuned to a mood of composed, relaxed serenity. A Da-sein is attuned to happiness if it is able to realize the potentialities essential to it.” (p. 211)

To put it more simply, Craig (1993), who collaborated with Boss from 1986 to 1989, summarized it as such:

“For Boss, the psychotherapist was first and foremost a human being whose most fundamental calling as such was to serve as a shepherd or servant of being and thus, in the practice of psychotherapy, to awaken the patient to his or her own-most possibilities for being-in-the-world.” (1993, p. 264)

May (1981) believed that the purpose of psychotherapy was to set people free. Existential psychologists describe freedom as the ability to choose within the limits, or existential givens, of life be they natural, such as death and separation or the limits established by humans such as culture and language (Schneider and Krug, 2017). Without acknowledging the limits of life, our relationship with those limits becomes problematic in either expansive or restrictive ways (Schneider and May, 1995). Denial or over identification with the limits of life, limits the possibilities within life. Without acknowledging the limits people may become power-hungry hedonists, eventually seeing others as non-player characters in their story on one polar end while an over-identification with the limits may a lead to a preoccupation with rules and unquestioning conformity on the other (Schneider and Krug, 2017).

Existential philosophers and their forefathers emphasize authenticity and confronting life on life's terms and doing so without self-deception. Kierkegaard, Nietzsche, Heidegger, Sartre, de Beauvoir, and many others believed that blind acceptance of the intellectual, cultural, and moral climate of the day either led to dysfunction or at the very least, limited the possibilities in life, or one's existentiality according to Heidegger.

In contemporary approaches such as Schneider's Existential-Integrative approach, four core aims are identified:

“(a) to help clients to become more present to themselves and others; (b) to help them experience the ways in which they both mobilize and block themselves from fuller presence; (c) to help them take responsibility for the construction of the current lives; and (d) to help them choose or actualize ways of being in their outside lives based on facing, not avoiding, the existential givens such as finiteness, ambiguity, and anxiety.” (Van Deurzen et al., 2019, p. 254)

This inner clarity, attunement to fundamental nature, and realization of potentials can be disrupted in many ways. Karl Jaspers introduced limit situations; and Yalom: lack/loss of freedom, isolation, meaninglessness and death. Varying approaches in existential work emphasize different approaches toward healing others as they encounter inevitabilities in life. Others may include sickness, anxiety, loss, interpersonal conflict, suffering, etc.

In light of these goals and the context of its history and its aims, the convergence between neuropsychological science and ideas in existentialism are ready to be approached.

### Time

Prior to our own intentions, we exist in a primordial relationship with time and space, and this relationship can be impacted by a myriad of challenging conditions. Psychologically, time may be examined in multiple ways and in relation to one another: subjectively (i.e., phenomenologically), objectively (i.e., clock time), and through mental time travel (i.e., autobiographical recall, prospection, present).

Of relevance, one of the primary networks in the brain related to self-processing is the Default Mode Network (Chan, 2016; Chan et al., 2024a,b), and this relates to mental time travel functions, as well as social cognition. In essence, this network ties together time and relationships, with the self. May (1963) defines the self as

“the organizing function within the individual and the function by means of which one human being can relate to another.” (p. 63)

In this context, May was accurate in understanding the self as an organizing function (e.g., self-referential processing), as well as bridging together others and the self; however what is missing in this definition (though he supports it elsewhere) is its fundamental relationship to time, and its existence as a process rather than an entity. Moreover, the self is really the result of an amalgamation of several processes (Chan, 2021).

French psychiatrist Minkowski (1970) explored the phenomenology of lived time to lived space which marries the inner world with the physical world. He was the first existential phenomenologist to describe how the disruption of temporal experience may be the foundations of psychological disturbance in schizophrenia. From Minkowski's perspective, all psychological disorders result from distortion in lived time and/or lived space. One demonstration of the consequences of the disconnection between time and space is presented by a depressive patient of Minkowski's who experiences a dislocation of present, past, and future. The patient reports experiencing life moment by moment, with the present feeling like an idea, the past feeling as an obsessive hallucination intruding on the present, and the future reduced to imagination. With the disintegration of perceived lived time, Minkowski further suggests that separation in the static and dynamic nature of time can lead to withdrawal from the present and the potential to hold onto regret. Van Deurzen et al. (2019) speaks to tensions in life, such as that displayed in Minkowski's patient who is presented with the challenge of living presently with acceptance of the past, as unavoidable. From this perspective, establishing a clear recognition of the roots of tension from the past is required to adequately embrace the present.

With Minkowski's (1970) approach, the concept of time and space is developed along with the human personality which is required to think of oneself in the future and develop a life history. With this ability, one can both establish and transcend individuality to act in harmony with their environment. Van Deurzen et al. (2019) explains that modern society is presented with a dilemma where we must compete to perform and produce to survive to the extent that we do not have time to enjoy the fruits of labor. To free ourselves from this dilemma, Minkowski believes we must reject the idea of time imposed by modern culture and transform thinking toward a way of living freely and spontaneously in time.

One temporal characteristic of existentialism is its emphasis on the future rather than the past. As May notes our memories become selective depending on how we see ourselves in the future, and our future that drives the present, not the past. The prominent researcher in Prospection, D'Argembeau (2021) summarizes

“The sense of self and identity can thus be fostered by the imagination of meaningful events that we anticipate to happen in the future; indeed, several studies have shown that future events are, on average, considered as more important to the self than past events.” (p. 276).

In fact, memories are known to be continually re-interpreted in the context of the present, usually informing some future behavior. This is at least partly how psychotherapy is effective. Troublesome memories are retrieved, and reconsolidated in a new light (Chan, 2021). This makes sense considering that memory traces recombine flexibly to form simulated future scenarios, further emphasizing, beyond our common understanding that memories are not reliable, that they are not for keeping a record of the past but for anticipating the future (Schacter et al., 2009).

A great deal of research has gone into the alterations in time and space, from the perspective of neuropsychology, such has been demonstrated by work pioneered by Georg Northoff. To begin with Northoffs work, termed the spatiotemporal approach, has found numerous accounts of how our brains spatiotemporal activity, defined by neural waves may, synchronize and de-synchronize with electromagnetic waves in the world; resulting in a variety of subjective experience corresponding to alterations in space and time. In Schizophrenia, as an example Northoff et al. (2021) encountered an

“abnormal prolongation in intrinsic neural timescale during self-reference in SCZ including its relation to basic self-disorder and negative symptoms. Our results point to abnormal relation of self and temporal integration at the core of SCZ.” (p. 170)

Indeed, May (1963) mentioned that in depression, the future may become “annihilated” (p. 140).

All other possibilities are collapsed, as the future is “emptied out.” In depression, Northoff and Hirjak (2024) also encountered the perception of time moving slower and the

“absence of changes due to the stalling of time in the past.” (p. 3)

Moreover in the depressed individual, there is typically abnormally slow activity in the sensory and motor cortices, as well as reductions of speed in the neural and affective cognitive strata.

Beyond the spatiotemporal approach, other models have begun identifying time as a foundational component that should be considered in both research and clinical work. As an example, Lanius and Frewen (2017), introduced a 4-dimensional model of trauma, one of which includes the experience of time. In particular, traumatized individuals may struggle to be in contact with the present, as they are feeling stuck in the past. Moreover, even victims with *covert trauma* (i.e., trauma without PTSD) may experience alterations in the quality of their ability to mental time travel (Chan et al., 2024a). Another clinical model is the temporospatial model (Chan et al., 2022) created to assist clinicians in tracking the moment-to-moment evolution of neurocognitive and affective activity ranging from sensation and emotion to abstract thinking.

### Space

“Dasein's ‘there,' its spatiality, is grounded in the fact that dasein is essentially world-disclosure” (Page 43, Boss, 1963).

This means that fundamentally, to exist is to always be accompanied by the active expression of a spatial world. How is this spatial world related to our brains? The three most evident lobes of interest are the occipital lobes (also known as the visual cortex), the parietal lobes, which play a role in visuospatial perception and mental imagery, and the temporal lobes which houses the ventral pathway, a path that is actively involved in helping us identify what objects are. Disruptions in relevant areas lead to a fragmented world-disclosure.

Northoff (2018) identified three nested hierarchies of space-time through electromagnetic frequencies within the brain. It is of no surprise that the deepest layer is entirely entwined with the world (what he calls the neuroecological layer). In his work, this relationship creates a layer of space-time that serves as the foundation required for inner experience. In fact, significant experiences in early childhood have been found to emit unique signatures that can be identified (such as in trauma, Northoff, 2016). More recently, there have been attempts to apply the foundations of inner experiences as an expression of relation between inner space-time and its resonance with outer space-time in a psychotherapeutic context (Chan et al., 2022). In this work, “spatial dilation” or the expansion of space within oneself can be considered a psychotherapeutic goal. This may manifest as the capacity to think in multiple dimensions. Generally, this appears as moving from an undifferentiated ground to an identification with pre-reflective thinking (i.e., length), to thinking as a self-differentiated from others (i.e., width) and thirdly into meta-cognition (i.e., height, top-down). These dimensions of space correspond well to Vandekerckhove and Panksepp (2009) elaboration of three phases of consciousness, each of which builds upon one another in ascending fashion. Anoetic consciousness relating to pure sensory and affective experience—related to brain stem activity. Second, noetic consciousness, related to a complex self-process. Here primary emotions elaborate into social emotions, alongside a pure process of thinking, without reflective capacity. Finally, we have meta-cognition, which allows us to think about thinking—which recruits cortical systems as they regulate subcortical regions (i.e., top-down).

Boss (1979) also points out that humans are a “world-spanning receptive realm of perception,” (p. 90) where psychological proximity and physical distance need not correspond. While I may be close to a particular person in space, I may still feel distanced from them, or in contrast I may be feel deeply connected to another person, even though they reside hundreds of miles away. This is partly why existential work emphasizes working within the “here and now” as a baseline, as it prevents psychic distancing, even if two people are in the same place. Finally, ideas such as repression may be re-interpreted as a reaction to difficulty such that the individual restricts access to inner space in contrast to the traditional Freudian view of it as a defense mechanism where distressing material is forced into the unconscious later to resurface as symptoms. Individuals thus structure their engagement with the world in such a way that it forecloses possibilities before they can be encountered, as a way to protect themselves from further suffering (Boss, 1979).

### Attunement

Attunement refers to how the world is disclosed to us at any given time due to the mood that we are in. The type of emotional experiences we undergo, opens the world to us in a particular way, at times narrowing our experience and other times expanding. These states allow possibilities for being to be experienced and fulfilled (Boss, 1979).

Attunement and attention go hand in hand. In neuropsychology, there are three systems of attention, the alerting, orienting and executive systems (Posner and Petersen, 1990). The alerting system which involves the reticular activating system and regions modulated by neurotransmitters like norepinephrine from the locus coeruleus, relates to arousal, it heightens our sensory awareness toward the world, much like a flood light. This pre-reflective system will thus open what we attend to. The orienting system shifts attention to focus on specific stimuli localized in space. This includes regions like the superior parietal lobule, intraparietal sulcus, and frontal eye fields. The temporoparietal junction and the ventral frontal cortex becomes active when anomalous activity arises, thus giving priority to some stimuli over others. Thus, allowing us to attend to the world in a particular way. Finally, the executive system is involved with conflict resolution, prioritization, and sustained attention and is primarily supported by the prefrontal cortex, including the dorsolateral prefrontal cortex, as well as the anterior cingulate cortex, posterior parietal cortex, and subcortical structures like the caudate nucleus. Attunement will shape how Dasein navigates competing demands within the world. Put simply, we have networks that allow Dasein readiness, direction and prioritization. Undergirding our attentional circuitry are our emotional systems. It is well known that emotional experiences have a direct connection to our attentional circuit (Yiend, 2010). Together, these concepts help form the basis of Attunement.

### Body

It is a misconception to think that existentialism is purely an abstract approach, when in fact one of its primary pillars is the importance of embodiment. Boss (1979) introduced the concept of “bodying forth” whereby the realization of potential is always expressed by extension of the body. We do not exist in a vacuum, isolated from the world, rather we are each expressions of the world through our bodies. Recent science has corroborated this perspective, with evidence supporting our gut as a center of emotion and intelligence, alongside our heart. Recent research in the field of trauma, has been extensively focused on how the “body keeps the score” or how the human vagus nerve plays a central role in dysregulation. Another domain of increased recognition relates to the “4E's of cognition,” more specifically meaning how cognition may be embodied, embedded, enacted, and extended. This is in resonance with the work of Merleau Ponty who describes humans as being “intertwined' with the world. Nietzche and Merleau-Ponty perceive the body as our connection to the world and shape our perceptions. The body has been widely recognized as an important consideration to the whole of therapy.

The gut-brain axis refers to the passage of information among the brain, gut, and gut microbiome. There are in fact more passageways from the gut to the brain, than the brain to the gut. Specific neurotransmitters and hormones play a modulatory role in the gut-brain axis, influencing gastrointestinal and mental health ailments. Recent studies show that individuals diagnosed with psychotic or affective disorders have altered gut microbiomes compared to healthy controls (Vindegaard et al., 2021). Alongside vagal fibers, the gut microbiome contributes to interoception that can have varying effects on neural processes (Mayer et al., 2022). Many neurotransmitters have precursors that travel from the gut to the brain. When gut microbiota is altered, it subsequently affects the level of adequate neurotransmitters in the brain such as serotonin or dopamine, leading to mood disorders including but not limited to anxiety and depression (Chen et al., 2021). Friedrich Nietzsche suggests that the physical body and its arousal play an integral part in identifying and interpreting affective states (Fowles, 2020; Nietzsche, 1973). This has been well documented in scientific literature.

According to Nietzsche, human existence is enhanced by illness and suffering, which plays a prominent role in how one interprets and relates to the world. Nietzsche states,

“The discipline of suffering, of great suffering—do you not know that it is this discipline alone which has created every elevation of mankind hitherto? (p. 115)”

Thus, the body becomes a vessel of transformation shaped by both internal and external signals which serve as catalysts for resilience and self-overcoming. Merleau-Ponty expresses his belief in these internal connections,

“Can I not find in the body some threads that the internal organs send to the brain that are instituted by nature in order to give the soul the opportunity to sense the body.” (p. 78)

The heart-brain axis is a contemporary example of an internal system in which dysregulation can lead to illness. Research shows that heart disease is linked to an elevated risk of developing dementia and overall cognitive decline (Simats et al., 2024). Dementia and cognitive decline can profoundly affect the quality of daily living as mediated through bodily functions. Further, cognitive impairment has been shown to occur after the onset of heart failure affecting various cognitive functions including but not limited to global cognition and verbal memory (Goh et al., 2022). Additionally, coronary heart disease is associated with psychiatric disorders, such as depression (Dammen et al., 2023). Disequilibrium amongst bodily functions, such as heart conditions, can have detrimental effects on other bodily systems.

Merleau-Ponty firmly asserts that the body is not simply a passive vessel for our mind. The body takes on an active role that can influence and be influenced by external factors. Merleau-Ponty states, “My organism is not like some inert thing, it itself sketches out the movement of existence (p. 86).” The body is capable of engaging with the world and subsequently interpreting what it receives. Internal pathways linking the brain and body also emphasize the role of the body in responding to psychological difficulties, as the body and mind are not separate, but thoroughly connected through consciousness. Merleau-Ponty emphasizes the brain and body connection, “The factual psyche, along with its “particularities,” was no longer an event in objective time and in the external world, but rather an event that we touched from the inside, of which we were the perpetual accomplishment or upsurge, and that continuously gathered together its past, its body and its world (p. 99).”

The gut-brain and heart-brain, as previously discussed, along with the central nervous system and vagal nerve, are all direct pathways from the body to the brain. The body is not only active within the world but also in relaying information to the brain. The feedforward system in the body refers to the bodily anticipation and performing of an action prior to consciousness awareness. Experience gained over time, sometimes decades, allows the body to have prepared itself to adapt and respond to changes in the environment. During this time, the body receives information from the environment and produces an automatic response driven by the spinal cord and motor neurons without sending or receiving information to and from the brain.

The lived experience of trauma does not limit its profound effects on one's mind. Adverse effects of trauma may present in various ways, affecting both the mind and body. This is because our bodies live through and internalize our experiences, similarly to how our brains process information. During childhood, exposure to traumatic events whether physical or verbal leads to alterations in brain composition including a reduction in gray matter in visual cortices and left auditory cortex and creates an overly sensitive amygdala, a risk factor for developing anxiety and depression (Teicher et al., 2022). Severe enough trauma, regardless of age, can pose significant health risks. Post traumatic stress disorder is considered a risk factor for developing cardiovascular disease and/or suffering a stroke (Perkins et al., 2021). Our bodies, not only our minds, respond to the impact of severe external stimuli. While experiencing a traumatic event, prolonged stress, or even a startling situation, the body has a physiological response.

Commonly known as the flight or fight response, the body experiences an influx of hormones and neurotransmitters that create an arousal response. Beneficial for short periods, significant or repeated activation of this pathway can alter physical and neurological processes. Van der Kolk (2015) discusses the internalization of the trauma response and its physiological manifestations. The body is the vessel that directly interacts and moves through the world, acting on predictions or reactions to the environment,

“... the consciousness of the body invades the body, the soul spreads across all of its parts, and behavior overflows its central region (Merleau-Ponty, p. 78).”

Upon receiving cues related to traumatic events, it is natural that the body emits a response whether internal, external, or both. Internally, heart rate can increase while externally, as the body itself is rooted in the physical world, posture can also change as a response to trauma evoking situations colloquially known as fight, flight, freeze responses (Ogden, 2021). Those who have experienced these events and develop a trauma response, whether due to war or assault, exhibit bodily effects (Van der Kolk, 2015). Related to Merleau-Ponty's idea, the body perceives and reacts to its surroundings. Treatment for these disorders also encompasses the mind and body to be effective. The mind and body are intricately connected with one another and the world regarding the adaptation to and internalization of external stimuli.

Some treatments take on a bottom-up approach and center around grounding techniques. These techniques focus on bringing an individual back into their body, or the present moment, by having them focus on bodily functions such as breathing. The Trauma Resiliency Model focuses on human resiliency and recognizing, understanding, and utilizing learning skills to control bodily sensations (Grabbe and Miller-Karas, 2018). Current literature supports the importance of including the body in treating psychological symptoms. Somatic experience may give rise to unwanted cognitions, and the same is true for the inverse. Van der Kolk (2015) also questions to what extent unbearable somatic experiences lead to negative coping strategies and poor mental health outcomes. Sensorimotor Psychotherapy, founded by Pat Odgen, takes on a holistic approach. This form of psychotherapy focuses on the somatic symptoms that arise from trauma and how they are best treated. Negative experiences that we face have a hand in shaping patterns of behavior and body posture as a result of repeated stimuli (Ogden, 2021). Rather than assume harmful coping mechanisms, Sensorimotor Psychotherapy focuses on the ability of the individual to adopt more resilient habits, including humor and self-awareness, that can be learned, as it is an innate characteristic (Buckley et al., 2018). The treatment goes beyond the mind. Merleau-Ponty states,

“I regard my body, which is my point of view upon the world, as one of the objects of that world.” (p. 81)

The Trauma Resiliency Model, Sensorimotor Psychotherapy, and other treatment approaches focus on bodily sensation and view the body as one's connection to the world. Known as embodiment, existential psychology posits that the body is fundamental in the ability to perceive, interact with, and be influenced by the environment. This view includes forming a deep understanding of the body in all states of health with the ability to acknowledge illness and suffering to better understand why they are present and how to treat them (Binder, 2022). A perpetual fact of the human condition is that discomfort and suffering will appear at various points throughout one's existence. Rather than avoiding this fact, spending time learning how to engage and accept the discomfort could lead to how to treat it. When experiencing physical or mental discomfort, is it necessary to understand the body's role in order to reduce suffering. For example, decreased physical movement is associated with a higher risk of developing cardiovascular disease, specifically remaining sedentary for 12 or more hours at a time (Ahmadi et al., 2024). Simply engaging the body in movement throughout the day can lower the risk of developing cardiovascular disease. The positioning of one's body can also have an effect on their creative thinking. Engaging in open body posture while in a positive emotional state has been shown to increase creativity (Hao et al., 2017). The body is an important mechanism allowing an individual to experience the world.

The body exists as an independent object within the world facilitating our interactions,

“I consider my body, which is my point of view upon the world, as one of the objects of that world.” (Merleau-Ponty, p. 73)

The COVID-19 pandemic is an example of a recent event demonstrating the concept that the body is an object within the world with the ability to enact change and is necessary to form actions and perceptions. During this time, it was imperative to quarantine to reduce the spread of illness. Unfortunately, isolating oneself from social interaction can have negative impacts. Depression, anxiety, and other mental health disorders were found to increase during the pandemic when social and, consequently, bodily interactions were at their lowest point (Rajkumar et al., 2022). Rajkumar et al. (2022) found increased sedentary behavior and decreased physical activity to be prominent risk factors for increased rates of mental illness, with at-home exercises and recreational activities being some of the main protective factors. Aligned with Merleau-Ponty's viewpoint, the COVID-19 pandemic demonstrated the physical body's critical role in mediating our relationship with the world and how that subsequently affects mental health.

### Relationship

The most obvious support for existentialism is the robust finding that the quality of the relationship exceeds the use of technique in all therapies (Wampold, 2015). Beyond the resonance of a relationship defined by unconditional positive regard, empathy and congruence (Rogers, 1951); it may be that the therapeutic alliance enables the actualization of strategies, techniques and ways of being that lead to enhanced wellbeing (Bordin, 1979; Steindl et al., 2023). Attachment research, and the general effects of human relationships on the brain are well documented (Siegel, 1999; Cozolino, 2024) demonstrating how *interwoven* humans are with one another. The mind is best understood as extending beyond the brain, into the body, environment and relationships (Chan, 2021).

Biologically, this is more greatly understood in the idea of *autopoesis* (literally self-production), a term introduced by Maturana and Varela (1980). This idea refers to living systems as *relational* networks of processes that are *self-generating* through the continual production of their boundaries and other components. In their own words

“living systems are units of interactions; they exist in an ambience. From a purely biological point of view they cannot be understood independently of that part of the ambience with which they interact…” (p.9)

Through this perspective, both the therapist and client can be understood as self-organizing, embodied systems whose identities are dynamically maintained and reshaped through their ongoing engagement with the world. The coupling between the therapist and client can generate a co-created field which may have its own impact on either or both systems. This is a greater idea of the relationship, one which is present in each individual, yet transcendent through synergy.^9^

Existential psychology and humanistic psychology's union formed around how both schools of thought viewed practitioner's relationships with those they treated. The relationship is viewed as an “honest, respectful relationship between equals” (Underwood, 2025). Martin Buber termed this way of relating as I-thou relationships that prizes the personhood of both the practitioner and client equally in a bidirectionality that leaves both parties impacted. While not all relationships can or should be I-thou relationships, I-thou relationships avoid agendas or any manipulation as opposed to I-it types of relationships where people view others as objects or other characters in their stories and interactions as transactional (Jourard, 1971; Yalom, 2002). This relationship requires the therapist to be authentic and perhaps vulnerable as opposed to feigning cordiality. It is well supported that the therapeutic alliance, the therapist as a person, the relationship itself and social and interpersonal context are more potent factors than medicalized and technical approaches in psychotherapy (Elkins, 2015).

During this era of technology, an additional dimension of experience is introduced to the social world. Van Deurzen et al. (2019) argues that although communication is readily available today, we are most at risk for alienation and endangerment of personal relationships. The consequences of alienation may also influence overall health and wellbeing, as a correlation is seen with the total quantity and quality of social relationships (Holt-Lunstad, 2017).

To contextualize the social element to the human experience, Van Deurzen et al. (2019) leans on the concept of *dasein* which draws on ideas from various philosophers, such as Heidegger who describe the study of one's embodied engagement to and within the world. Heidegger emphasizes the relationship between inherent culture and the time we are born into, which influences our personal and social experiences. With today's technology and culture of social media, fostering social relationships may look different but it is unclear if a lasting connection can be sustained by solely relying on these devices. As tools like artificial intelligence (AI) are used more frequently by consumers, the social relationship between a human and AI may also shed light on the future of one's engagement with the social world.

### Freedom

Kirk Schneider's existential-integrative approach frames freedom as a dynamic balance between constriction and expansion, where individuals often retreat into rigid certainty or unstructured openness to avoid the groundlessness (e.g., chaos or obliteration) that, through trauma, associates with either extreme expansion or extreme constriction. True freedom, he argues, emerges from integrating these polarities, as

“these two polar eventualities—chaos and obliteration, greatness and smallness—haunt the entire spectrum of freedoms.” (p. 40, 2008)

Rather than escaping these tensions, he advocates for

“careful and sensitive therapeutic invitations to stay present to denied constrictive or expansive parts of self.” (p. 40, 2008)

A key tool in this process is the “lens of vastness,” which expands awareness beyond self-imposed limitations. Schneider describes it as opening

“in infinite directions… in the distances and horizons, in parks, and in planetariums, in the chemical make-up of matter, and amid the diversity of species.” (Schneider, 2019, p. 125, Schneider and Krug, 2017)

This broader perspective disrupts existential constriction by loosening entrenched self-focus and habitual thought loops that reinforce fear and stagnation.

Neuroscientific findings from Van Elk et al. (2019) support Schneider (2009, 2019, 2023) view of awe as a gateway to existential depth, showing that it weakens activity in the default mode network (DMN), specifically the medial prefrontal cortex and posterior cingulate cortex, which regulate self-referential cognition and autobiographical processing. As a result, awe momentarily suspends rigid identity structures, allowing individuals to experience a sense of transcendence. Simultaneously, awe activates the frontoparietal network, including the dorsolateral prefrontal cortex and inferior parietal lobule, areas responsible for attentional flexibility, executive control, and perspective-taking. This dual effect: suppressing DMN activity while engaging the frontoparietal network, shows how psychological expansion is necessary for existential freedom. Thus, awe serves as a neurocognitive bridge between existential constriction and experiential liberation. Awe is indeed considered a self-transcendent experience, and a *vital signal* that may direct humans toward more meaningful activity, and that induces an effect not limited to inner life but to the body as well (Chan, 2021).

This neurocognitive shift supports Schneider's view that true freedom emerges from engaging with both vastness and intricacy.

“If the lens of vastness orients us to the macro and grand, the lens of intricacy situates us in the micro and hidden... The more one is informed by such engagements, the greater one's potential for a full and diversified life—a life of depth but also vibrancy” (Schneider, 2019, p. 125)

Rather than being trapped in rigid expansion or constriction, freedom lies in fluidly moving between these states, embracing life's complexity without being consumed by existential dread.

Awe, by temporarily suspending habitual self-focus, offers a pathway to this dynamic freedom. Awe-based interventions such as guided nature experiences, contemplative practices, and engagement with vast artistic or philosophical ideas act as a neural reset, fostering cognitive flexibility and openness to new possibilities. Schneider (2019) describes this as the ability to “seize upon the ‘blank canvas' of our day,” stepping beyond entrenched patterns to

“breathe a little freer, a little fuller, wherever we are” (p. 124)

Integrated into therapy through mindfulness, immersive environments, and guided reflection, awe helps individuals break free from existential stagnation, cultivating the psychological flexibility necessary to engage with life's uncertainties while maintaining a sense of agency and meaning.

The concept of transcendence is also of interest. According Jaspers (1971)

“Transcendence is the power through which I am myself: where I am truly free, it is precisely because of transcendence. Its most decisive language is the one which speaks through my Freedom” (p. 80)

Transcendence is not about denying the world, but to integrate and foster our potential within it. It is a *power* within us that pushes us to think beyond our limitations, that lures us into its encompassing horizon.

While free will continues to be hotly debated, there exist many compatibilist and incompatibilist models on both ends of the spectrum. Recent models in science view humans as complex systems composed of hierarchical processes of information (e.g., biology, sensations, emotions, beliefs, thoughts, etc.) capable of transcending their input and creating changes at lower levels. This is called recursion, which is a property of complex systems that enables a process to change that from which it comes from (Chan, 2021). This is reflected in the idea of downward causality in neural networks, where higher order neural states can influence and regulate lower-level neural processes. The mindbrain is non-linear, which means that the argument that just because biological systems *may* precede the emergence of consciousness does not mean that our consciousness is determined by our biology (that would be linear thinking). Non-linearity means that a “cause” can have as much “effect” on the “effect” as does the “effect” on the “cause” which can be found in living systems (i.e., closed-loop causation vs. open loop causation, Tse, 2024).

A thorough and vicious argument for libertarian free will within a physicalist framework can be found in the work of Tse (2013, 2024). Tse (2013) coined the term “criterial causation” to describe how synapses can be “reweighted” to change criteria for future decisions (e.g., on when to and when not to fire). These top-down influences open the potential for a degree of freedom in choice. As Jaspers (1971) notes

“existenz is the self-being that relates to itself and thereby also to transcendence from which it knows that it has been given to itself and upon which it is rounded.” (p. 21)

Simone de Beauvoir's existential ethics presents freedom as an ongoing negotiation between facticity (given conditions) and transcendence (self-creation through action). Unlike Sartre's radical autonomy, she argues that freedom is interdependent and must be exercised through ethical engagement with others. Ethical action is never absolute but must be actively constructed in response to situational constraints and evolving contexts. Neuroscientific findings support this, showing that moral reasoning uniquely engages the DMN, which facilitates perspective-taking and self-other integration, reinforcing Beauvoir's claim that true freedom requires recognizing others as equally free (Ambrase et al., 2024). Furthermore, ethical decision-making under ambiguity activates core nodes of the salience network, particularly the right anterior insula (aINS), dorsal anterior cingulate cortex (dACC), and right medial frontal gyrus (MFG), which are involved in detecting uncertainty, processing moral salience, and guiding adaptive decision-making (Ambrase et al., 2024).

Ambiguity-specific decision-making uniquely engages subcortical structures such as the thalamus and midbrain, which are key components of the cortico-striato-thalamo-cortical loop. Unlike risk-based decision-making, which engages the frontoparietal network (middle frontal gyrus, lateral occipital cortex, and intraparietal sulcus) for structured cost-benefit analysis, ambiguity processing does not rely on explicit probability calculations. Instead, it requires greater reliance on the salience network and midbrain systems for detecting uncertainty and adapting behavior, supporting Beauvoir's idea that ethical action is shaped by navigating the unknown rather than deferring to rigid structures. The convergence of the aINS, dACC, and thalamus in ambiguity-based decision-making suggests that ethical choices are not merely about evaluating probabilities but about confronting the limits of knowledge and acting within uncertainty, mirroring Beauvoir's existentialist ethics.

Conversely, studies also suggest that when moral reasoning becomes habitual, reliance on these cognitive control regions diminishes, implying that ethical engagement can transition from a deliberative to an intuitive process when deeply internalized (Cherry et al., 2015). For beauvoir, freedom is action-oriented: “Freedom realizes itself only by engaging itself in the world: to such an extent that man's project toward freedom is embodied for him in definite acts of behavior.” This perspective aligns with research showing that goal-directed behavior in ethical contexts is shaped by dopaminergic motivation systems, while emotional and social dimensions of moral reasoning are mediated by the vmPFC and anterior insula (aINS), which integrate affective responses into decision-making (Ambrase et al., 2024; Hopkins and Deepa, 2018). Higher emotional intelligence has been found to enhance ethical engagement, as individuals with stronger emotional awareness and regulation show increased ethical sensitivity and a greater ability to balance competing moral values (Hopkins and Deepa, 2018). The relationship between ambiguity tolerance, emotional affect, and ethical decision-making further reinforces that ethical freedom must be actively willed into existence rather than passively received.

Moardi et al. (2016) found that ethical decisiveness in management students was driven more by emotional sensitivity than cognitive flexibility, with stronger negative affect increasing moral commitment. In contrast, accounting students, who rely on structured, rule-based reasoning, showed no link between ambiguity tolerance and ethical decision-making. This aligns with Beauvoir's critique of the “serious man,” who clings to external moral systems rather than engaging with ethical ambiguity. Neuroscientific findings further support this distinction, showing that ambiguity-related moral reasoning activates the anterior cingulate cortex and dorsolateral prefrontal cortex, which facilitate cognitive control and conflict resolution, while emotionally engaged moral reasoning recruits the ventromedial prefrontal cortex and anterior insula, which integrate affective salience into ethical decision-making (Ambrase et al., 2024). Additionally, moral choices with greater social or personal impact activate reward-related structures such as the caudate nucleus (CN) and nucleus accumbens (NAcc), reinforcing the idea that ethical action is not a purely rational cost-benefit analysis but an embodied, value-driven process. As Beauvoir saw, freedom and moral responsibility are continuous acts of engagement shaped by both cognitive flexibility and emotional attunement to ethical stakes (Ambrase et al., 2024; Moardi et al., 2016).

## Courage

It was foundational to Heidegger's and Sartre's philosophies (Sartre, 2007) that we are beings concerned with our own being. To “be” in the existential sense is to take a stand on what *kind* of being you are, which is a matter of the meaning you make of your life. To be a vegetarian, for example, is to value animal wellbeing over their culinary uses, to be a revolutionary is to prefer a chance at a new world over preserving the old one. It is also central to the existentialist tradition that this very concern for one's being generates anxiety. Our anxiety over being is a pervasive and inescapable feature of life that cannot be eliminated even under conditions of perfect safety and plentiful resources. But as Tillich (2014) emphasized, there is also a courageous aspect to being. While not all courage is existential, since not all fear is existential (e.g., the courage to face one's fear of bridges in exposure therapy is not existential courage, for example), existential courage is courage in the face of existential anxiety. It is the courage to-be, and it is the courage to not-be.

The ultimate source of our anxiety to-be is groundlessness (absurdity), of which we can speak of two types: ontological groundlessness and normative groundlessness. Ontological groundlessness is the fact that there is nothing in our *nature* that determines facts about the kinds of people we are, so there is nothing more to who we are than what we do. Normative groundlessness is the fact that there are no objective facts about what kind of person anyone is *destined* to be or is *supposed* to be, and so our choices, in the final analysis, can never be justified, even to ourselves. Both types of groundlessness generate anxiety because they entail that questions about who one is and who one should be have no answers to discover. Instead, answers must be *invented* or *quested after*.

This understanding of courage can inform how we think of self-esteem and responsibility. If a person's self-esteem arises from a correspondence between their self-conception and their values, then low self-esteem arises when the gap between these two concepts becomes too wide, and restoring self-esteem becomes a matter of restoring the correspondence. Our values are intrinsically linked with our emotions, where emotions can be used to indicate our current position to our values. Van Deurzen et al. (2019) emotional compass illustrates the nature of reflection on our values and purposes being a critical practice in existential psychology, as well as embracing the consequences of our actions. According to this model, one is most aligned with their values, they are most likely to experience wellbeing; conversely, when one is disconnected they are likely unhappy. Fear can also indicate a person's transition away from their values and even manifest as disappointment of unfulfilled potential. Van Deurzen's work also highlights that a meaningful life requires personal limits to be defined and possibilities of life to be celebrated.

Groundlessness entails that the correspondence is not achievable by discovering something inside yourself or about yourself to take pride in, but by having the courage to be the kind of person you admire by acting accordingly. In this respect, decision making is crucial to the process of embracing groundlessness. To embody values-based behavior, consideration of choices, values, and consequences is essential to produce authentic decisions that reflect one's self concept. In order for groundlessness to occur, one must regulate cognitive dissonance and manage values-based decisions. Cognitive dissonance can arise when one's behavior contradicts their values, producing discomfort and emotions such as envy, shame, and sorrow. To reduce internal conflict, activity in the posterior medial and dorsolateral prefrontal cortex, nucleus accumbens, and posterior cingulate cortex helps detect conflict, managing values-based decisions, and anticipating future outcome (Tandetnik et al., 2021). Courage may play a role in minimizing cognitive dissonance and preventing non-adherence to these mechanisms to reduce internal conflict and preserve self-esteem.

While he is not concerned directly with self-esteem, Viktor Frankl is working with a similar understanding of groundlessness and the human condition when he says of his existential therapeutic approach logotherapy that

“[it] tries to make the patient fully aware of his own responsibleness; therefore, it must leave to him the option for what, to what, or to whom he understands himself to be responsible.” (Frankl, 2006 p. 109–110)

Likewise, he emphasizes that this responsibleness is not toward anything inside oneself, but

“by declaring that man is responsible and must actualize the potential meaning of his life, I wish to stress that the true meaning of life is to be discovered in the world rather than within man or his own psyche, as though it were a closed system.” (Frankl, 2006 p. 110)

The other aspect of existential courage is courage in the face of non-being. Whereas groundlessness is the source of our anxiety to-be, our anxiety over non-being stems from threats to—and the potential loss of—our being. The most obvious example of such a threat is our future death, and so the courage to not-be is, in one guise, the courage to pursue a meaningful life in spite our mortality. The courage to not-be is, however, a more general requirement of an authentic life because non-being always accompanies being; to be something is always thereby to also not-be something else.

That being is always accompanied by and stands in relief against non-being is a consequence of our facticity: including the facts that only a limited number of concrete opportunities present themselves to us at any given moment, and that we live in constantly flowing time and therefore do not have the option to seize every opportunity. As Frankl puts it,

“man constantly makes his choice concerning the mass of present potentialities; which of these will be condemned to nonbeing and which will be actualized?” (Frankl, 2006 p. 121)

Different life-options oppose each other, sometimes by embodying conflicting values, but also by literally taking up the limited time required to live any of them. We can therefore only fully understand the meaning of someone's action, and the kind of person they thereby are, against the backdrop of what they've decided *not* to do. The significance of what they do is partly determined by the significance of the alternatives they left unrealized.

We can see both types of courage at play in Sartre's story of the French student who came to him for advice while Paris was occupied by the Nazis (Sartre 1946, p. 30). The student's brother had been killed in the Nazi invasion, and he yearns to join the French resistance. However, were he to be killed, he would be leaving his mother entirely alone in life, and he also wants to be a loyal son to her. The student has the courage to make something meaningful of his exceedingly difficult situation, but his courage to-be also generates an agonizing choice. In this case, the student is not agonizing about being a patriot or a dutiful son, he is agonizing about *not* being one or the other. To be one, he must accept not being the other; the courage to-be also requires the courage to not-be.

That non-being is a fundamental condition of being means it cannot be avoided and anxiety over non-being is a permanent feature of the courageous individual.

“Courage is self-affirmation ‘in-spite of,' namely, in spite of nonbeing,” according to Tillich (2014), p. 66

“He who acts courageously takes, in his self-affirmation, the anxiety of nonbeing upon himself.” Tillich (2014), p. 66

This suggests that the courage to not-be requires, or, in some sense is, a type of acceptance. It is the acceptance of our own finitude and the willingness to affirm our own finitude enables us to affirm the whole of our being, including our positive being. When a person lacks the courage to not-be, the result is either despair, when they cannot affirm themselves, or, to avoid despair, neurosis. “*Neurosis is the way of avoiding nonbeing by avoiding being*” (Tillich, 2014, p. 66, italics in original), for, if one cannot accept non-being, then one cannot actualize one's potential being, either, and must deny one's potential being.

In relation to courage, one important region to consider is the anterior midcingulate cortex (aMCC). Located in the temporal lobes, the aMCC receives information and calculates resources needed to meet one's needs. It does so by adjusting to prediction errors, predicting behavioral outcomes, assessing energetic cost of opportunity, and monitoring and modulating the internal state of the body to prepare for action (Touroutoglou et al., 2020). In other words the aMCC is a precursor to “grit,” which may be understood as the ability to persist in the face of challenge toward ones goals. Spunt et al. (2012) also account that the aMCC region is positively correlated with a subjective sense of frustration during a difficult task (p. 1759). An individual's variability in courage is dependent on their subjective assessment of energetic cost of opportunity supporting or restricting their actions toward their courage-to-be or courage-not-to-be.

Underlying the idea of courage, is the ability to confront an event that elicits anxiety or fear.

Anxiety manifests when being is threatened. For Sartre, the Self is not only inherent in nature but in constant creation through its actions (Zhao, 2025). A person's choices and actions can influence the directionality for their future, but if this is lost, anxiety may ensue. According to Soren Kierkegaard anxiety is rooted in freedom and choice amongst a dizzying array of possibilities, it is a necessary experience that can be productively expressed. These conceptions can be related to our understanding of stress and anxiety. There is a substantial overlap in the neural circuits of stress and anxiety. Yerkes-Dodson Law refers to the inverted U shaped curve that demonstrates how mild-moderate levels of stress (i.e., eustress) can heighten cognitive performance. The benefits of increased stress in one's environment include better “cognitive function, attention, and memory retrieval” which helps the learning process (Cordova et al., 2023). It is also well known that neuroplasticity can be triggered by such events leading to growth. In this context, anxiety, as an awareness of freedom may optimize the recruitment of brain functioning that facilitates creative self-actualization.

These studies suggest while some degree of stress or specific stress hormones might benefit certain aspects of learning and memory, the relationship is complex and not consistently positive. High levels of anxiety can be detrimental, and the timing and duration of stress, as well as the type of stress, appear to influence the outcome.

## Conclusion

The purpose of this article is to begin the process of bridging the ontological with the ontic. While science and existentialism appear to differ at face value, a deeper analysis reveals that congruence can be found within these two seemingly disparate frameworks. In the quest for truth, science adopts the correspondence theory of truth, whereas philosophy careens toward the coherence theory of truth. Future comparative analysis focusing on incongruences between ideas in existentialism and facts from scientific investigation may lead to a mutual enrichment. Existential approaches have been criticized as being too unstructured; neuropsychology may be used to help *guide*^10^ the process of existential based interventions. Finding resonance between correspondence and coherence forms of truth would be wise. Some therapies like this already exist, such as Schneiders existential-integrative approach, and more recently the neurodaonamic^11^ approach introduced by Chan (2025).

In contrast, existential work may also benefit the natural sciences. Firstly, it helps maintain integrity and balance by questioning pre-existing assumptions made by the scientific community. In this vein, it prevents dogmatism and attempts to minimize biases. Secondly, it helps expand the boundaries of scientific inquiry when they are too narrow, and help demarcate boundaries when inquiry is too speculative and unfocused. Thirdly, by embracing imaginative ideas that engage with human experience it may inspire new hypotheses and deepen the contextual relevance of scientific advancement.

## Footnotes

1 The Neuroexistential approach, will be strictly discussed in a psychotherapeutic context, and has no relationship to the work of Caruso and Flanagan (2018).

2 It is important to distinguish the term *existential* from the term *existentialism*. The term *existential* as such refers to human existence as such, as it is lived in the raw, naked and the face of our having been “thrown” into the world into a time, a space a culture and a whole historical context not of our own making or choosing. It refers to the fact that we are the kind of being we are, one *who* lives with an awareness of our life and death, our finitude, our suffering, and all that comes with the mangy magnificence of being human. On the other hand, the term *existentialism* was not coined until the mid 1940s by the catholic philosopher and novelist Gabriel Marcel (1889–1973) referring to the work of Jean Paul Sartre (1905–1980). It refers to a *system of thought, an ideology or movement* that became especially fashionable in France during the 40s. However, as Soren Kierkegaard, widely regarded as the first “thinker of existence,” wrote that “an existential system is impossible” (Kierkegaard, 1941/1846, p. 107). He was not alone in this view. Only Simone de Beauvoir (1908–1986) and Sartre himself, identified as existentialists, every other thinker commonly thought to be an existentialist refused the label.

3 Intentionality refers to the circumstance that every mental act, whether a thought, feeling, or belief, is directed at an object, in other words, all conscious acts take an object and can only be understood with reference to them.

4 Sadly, it was not until 2018 at the American Psychological Association approved of the inclusion of qualitative studies in its publications.

5 Our particular concern here is with the physical nervous system, especially the brain.

6 Binswanger and Medard Boss add the distinct privilege of knowing both Freud and Heidegger (not to mention Bleuler and Jung).

7 Ontological may be understood as the study of the fundamental “structures,” conditions and experience of being. A textbook ontological question is “What does it mean to exist?” Ontic refers to the empirical world, and that which is measurable, factual, and concrete. It concerns particular beings and their qualities. An ontic question might be “What effects does depression have on the brain?”

8 In the field of scientific inquiry, the neurophenomenological approach is worth noting. This framework, championed by Varela et al. (1991), synthesized phenomenology, with objective scientific investigation and eastern meditation practices. It has gained in popularity and opened a space in scientific investigation to retain the humanity that is often lost in statistics with the hopes of also improving scientific rigor by reducing interpretive errors on account of personal biases.

9 In relationship to this idea, it will be of interest to begin integrating other perspectives including complex systems theory, predictive processing and the free energy principle to different existential frameworks. As an example, Längle's and Frankl's Logotherapy has recently been integrated through predictive processing (Leidig, 2025).

10 A note of parsimonious caution is warranted here. It is important not to make premature inferences from brain findings to complex human experiences and behaviors. Studies are not at the point where there is a 1–1 mapping, and extrapolations must respect the limits of science and the irreducible nature of existence.

11 A contemplative existential meta-framework integrating classical daoist philosophy with neuropsychology and evolutionary theory. Chan (2025) argues that beyond evolutionary mismatch, humanity is also faced with a dispositional mismatch. This is defined by his SAD theory and a disorientation to seven principles of nature
